# Development of sensitive and accurate solid-phase microextraction procedure for preconcentration of As(III) ions in real samples

**DOI:** 10.1038/s41598-021-84819-0

**Published:** 2021-03-09

**Authors:** Adil Elik, Mustafa Tuzen, Baki Hazer, Savaş Kaya, K. P. Katin, Nail Altunay

**Affiliations:** 1 Department of Chemistry, Sivas Cumhuriyet University, 58140 Sivas, Turkey; 2 Faculty of Science and Arts, Chemistry Department, Tokat Gaziosmanpasa University, 60250 Tokat, Turkey; 3 Center for Environment and Water, King Fahd University of Petroleum and Minerals, Research Institute, Dhahran, 31261 Saudi Arabia; 4 Department of Aircraft Airframe Engine Maintenance, Kapadokya University, Urgup, 50420 Nevşehir, Turkey; 5 Chemistry Department, Zonguldak Bulent Ecevit University, 67100 Zonguldak, Turkey; 6 Health Services Vocational School, Department of Pharmacy, Sivas Cumhuriyet University, 58140 Sivas, Turkey; 7 Institute of Nanoengineering in Electronics, Spintronics and Photonics, National Research Nuclear University “MEPhI”, Kashirskoe Shosse 31, Moscow, 115409 Russia; 8 Department of Biochemistry, Sivas Cumhuriyet University, TR-58140 Sivas, Turkey

**Keywords:** Environmental monitoring, Solid-phase synthesis

## Abstract

We synthesized the poly(methyl methacrylate-co-2-aminoethyl methacrylate (PMaema) amphiphilic copolymer in a form of solid phase adsorbent. Then it was used for separation, preconcentration and determination of trace amount of As(III) ions from foods and waters with hydride generation atomic absorption spectrometry. The PMaema was characterized by fourier transform infrared spectrometer and nuclear magnetic resonance spectrometer. The adsorption of As(III) to the PMaema was also supported using computational chemistry studies. The experimental parameters (pH, PMaema amount, adsorption time and ethanol volume) were optimized using a three-level Box–Behnken design with four experimental factors. We observed linear calibration curve for the PMaema amount in the 10–500 ng L^−1^ range (R^2^ = 0.9956). Limit of detection, preconcentration factor and sorbent capacity of PMaema were equal to 3.3 ng L^−1^, 100 and 75.8 mg g^−1^, respectively. The average recoveries (spiked at 50 ng L^−1^) changes in the range of 91.5–98.6% with acceptable relative standard deviation less than 4.3%. After validation studies, the method was successfully applied for separation, preconcentration and determination of trace amount of As(III) from foods and waters.

## Introduction

Arsenic is known as the toxic element for living organisms even at the ultra-trace levels. It leads to many human health problems, including skin and lung cancer^1^. Irrigation of soil, vegetables and plant crops with arsenic-contaminated water results in accumulation of arsenic in the plants^2^. Then it enters the human bodies through their daily diet.

Arsenic possesses different oxidation states in water, the most common of them are arsenite (As(III)) and arsenate (As(V)). Toxicity of arsenite is higher than arsenate^3,4^. However, arsenic does not have any health benefits either in the arsenite or arsenate forms. This is why it was commonly quantified as the total arsenic level rather than the fractions of individual species^1^. Determination of arsenic in water, food and environmental samples attracts great research interest due to its toxic effects on human and animal health^5,6^. The European Food Safety Authority proposed maximum permissible levels of inorganic arsenic of 100, 200, 250, and 300 μg kg^−1^ for rice cakes, polished and white rice, parboiled rice, wafer cookies, and rice for the production of foods for infants and children, respectively^7,8^. Environmental Protection Agency reduced the permissible standard of arsenic concentration in drinking water from 50 to 10 μg L^−1^^9^. According to the Turkish Food Codex, the level of arsenic in soft drinks may not exceed 0.1 μg g^−1^^10^.

Various instrumental analytical techniques, such as capillary electrophoresis^11^, inductively coupled plasma mass spectrometry (ICP-MS)^12^, electrothermal atomic absorption spectrometry (ETAAS)^13–15^, inductively coupled plasma optic emission spectrometry (ICP-OES)^16^, UV-spectrophotometer^17^ and hydride generation atomic absorption spectrometry (HGAAS)^18^ are widely used for determination of arsenic in water, food and environmental samples. The HGAAS has some advantages due to its low cost, high sensitivity and reproducibility. To detect arsenic in very low (trace and ultra-trace) concentrations in water and food samples using HGAAS, separation and preconcentration steps are necessary. Various separation and preconcentration methods, such as ultrasonic-assisted micro solid phase extraction^19^, hollow fiber liquid phase microextraction^20^, dispersive liquid–liquid microextraction^21^, enzyme based hydrolytic water phase microextraction method^22^, and solid phase microextraction^23^ have been proposed for separation and preconcentration of arsenic in different matrices. The advantages of solid phase microextraction over other preconcentration methods are its simplicity, high rate, short extraction time and high preconcentration factor. In addition, the efficiency of solid phase microextraction can be increased by novel recently proposed methods, including surfactant-assisted^24^ and ultrasonic-assisted^25^ preconcentration and using of N-doped mesoporous carbon-based^26^ and magnetized-based^27^ materials. These approaches provide faster phase separation during solid phase microextraction.

Recently, we used polystyrene-polydimethyl siloxane hydrophobic copolymer for separation and preconcentration of arsenic ions^28^. In this work, we used a new amphiphilic copolymer with pendant aminoethyl groups. Amphiphilic copolymers contain both hydrophilic and hydrophobic blocks^29,30^. Poly (methyl methacrylate) is a glassy, hydrophobic polymer. Poly (2-aminoethyl methacrylate) (PMaema) hydrochloride is a hydrophilic polymer, which belongs to a promise for gene delivery class of polymers^31^. The 2-aminoethyl methacrylate (Maema) can be (homo/co)polymerized by atom transfer radical polymerization which is a type of controlled living free radical polymerization^32,33^. In this study, Maema was copolymerized with hydrophobic MMA to decrease water solubility. Then, the amphiphilic copolymer poly (methyl methacrylate-co-2-aminoethyl methacrylate) was prepared via conventional free radical polymerization^34^. Prepared copolymer swelled but did not solute in water. We characterized this new copolymer (PMaema) and used it for separation, preconcentration and determination of arsenic ions in water and some food samples.

## Results

### FTIR and 1H NMR characterization of PMaema

**Scheme 1:**
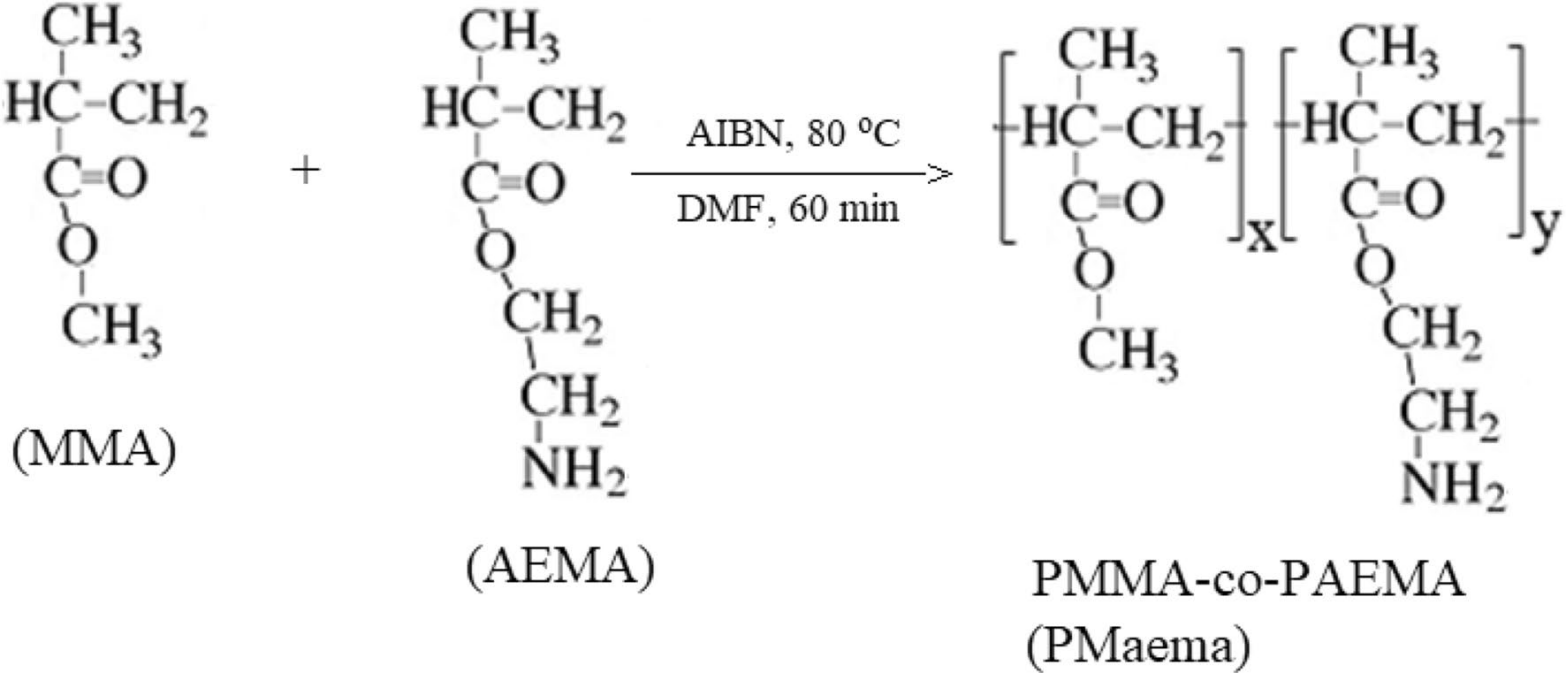
Reaction design of free radical copolymerization of MMA and AEMA in DMF solution.

Scheme 1 presents the polymerization reaction yielding to amine functionalized amphiphilic copolymer PMaema.

**Figure 1:**
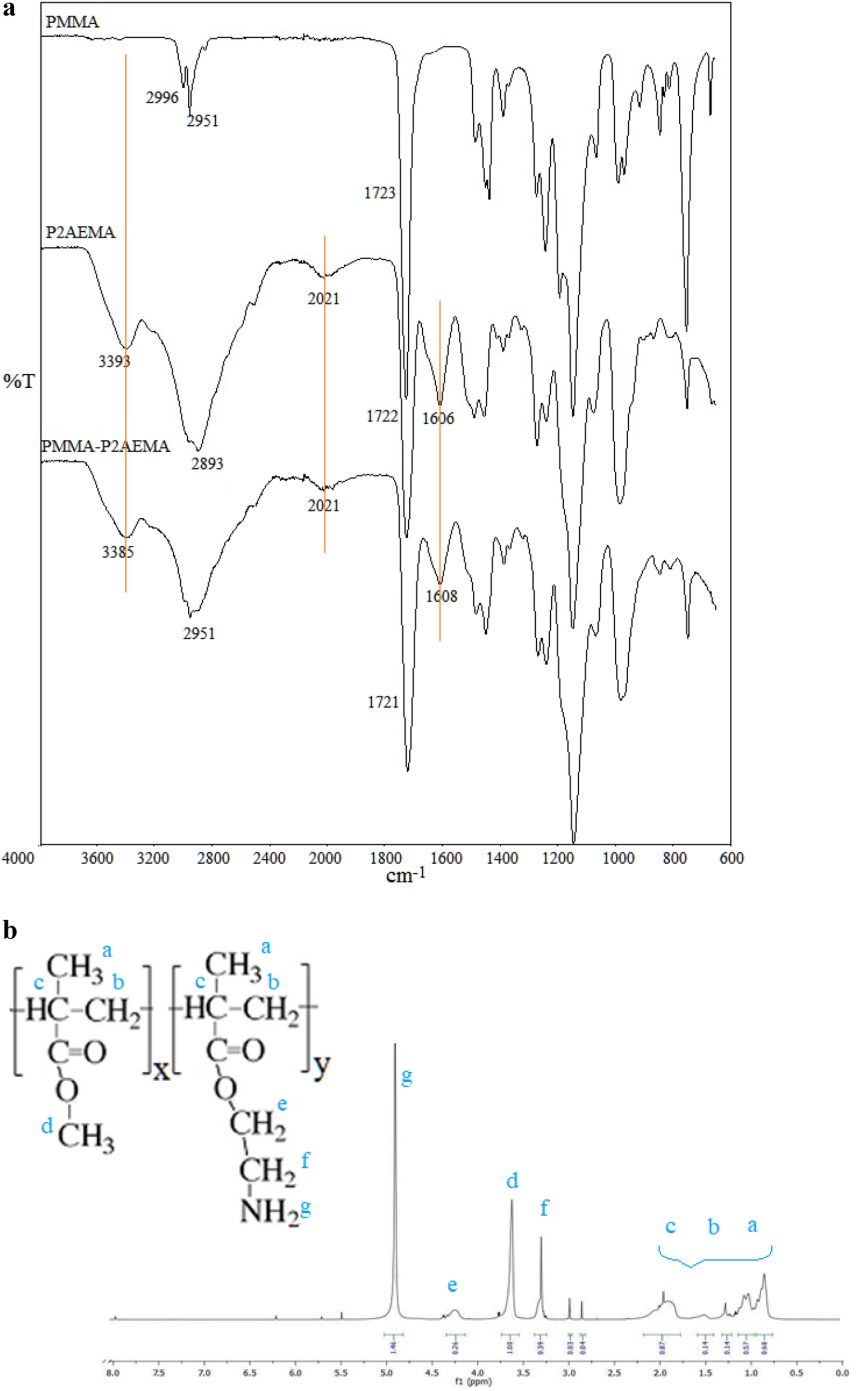
(**a**) FTIR spectra of PMMA, P2AEMA and Poly(MMA-co-AEMA). (**b**) ^1^H NMR spectrum of the amine functionalized amphiphilic copolymer (PMaema).

The characteristic signals of the PMaema copolymer were observed in both FTIR and ^1^H NMR spectra (Fig. 1a,b). They demonstrated good agreement with the previously reported spectra^35^. FTIR spectrum possessed peaks at 3410, 2021, 1606 and 1608 cm^−1^ (correspond to primary amine of aeMA) and at 1722 cm^−1^ (corresponds to carbonyl of MMA and aeMA). Figure 1a presents the comparative FTIR spectra of the amphiphilic copolymer with the related homopolymers. The characteristic signals of the copolymer can be clearly seen in this comparative FTIR spectra. ^1^H NMR spectra demonstrated chemical shifts of 4.95, 4.25, 3.65, 3.30 and 0.80/2.00 ppm, corresponding to –N**H**_2_ of aeMA, –C**H**_2_–O–, –OC**H**_3_, –C**H**_2_–NH_2_ and –C**H**_2_–C(C**H**_3_)– groups, respectively. Ratio of the integral values of the signals at 3.65 and 3.30 ppm shown that the molar concentration of PMaema copolymer in the aema was equal to 29% (see Fig. 1b).

### Computational chemistry approach

**Figure 2:**
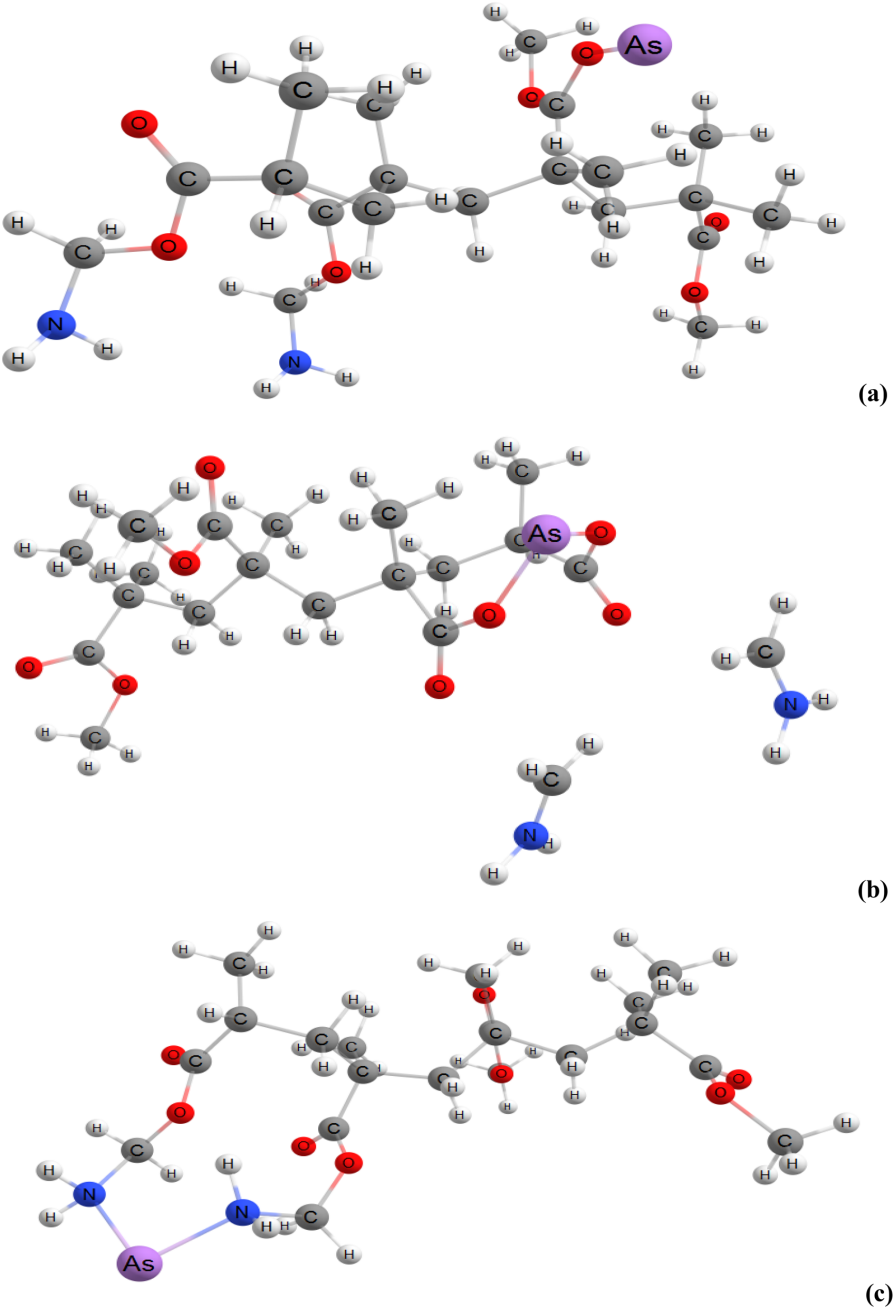
(**a**–**c**) Optimized structures of the As(III)-C_20_H_36_N_2_O_8_ complexes.

In our computational study, the PMaema copolymer was represented by the C_20_H_36_N_2_O_8_ molecule. We considered three possible geometries of As(III)-C_20_H_36_N_2_O_8_ (see Fig. 2a–c). Their characteristics are collected in the Table 1. The adsorption energies between As(III) ion and C_20_H_36_N_2_O_8_ molecule were calculated as *E*_b_ = *E*(C_20_H_36_N_2_O_8_) + *E*(As(III)) – *E*(As(III) − C_20_H_36_N_2_O_8_. The geometry presented at Fig. 2b provides the strongest adsorption. In this complex, nitrogen does not interact with the As(III) ion; moreover we observed detachment of the CH_2_-NH_2_ ligands. However, the role of amino groups is very important: they provide parallel displacement of the nearest PMaema fragments. Therefore, As(III) ion can interact simultaneously with two oxygen atoms (see Fig. 2b).

**Table 1:** Calculated characteristics of the C_20_H_36_N_2_O_8_ molecule and its complexes with the As(III) ion.

System	Charge (|*e*|)	*E*_b_ (eV)	HOMO (eV)	LUMO (eV)	Gap (eV)	Bonds lengths (Å)
C_20_H_36_N_2_O_8_	0	–	− 7.15	− 0.39	6.76	–
As(III)–C_20_H_36_N_2_O_8_ **(a)**	3	9.56	− 7.70	− 7.20	0.50	*l*_As–O_ = 1.781
As(III)–C_20_H_36_N_2_O_8_ **(b)**	3	14.17	− 7.81	− 4.98	2.83	*l*_As–O1_ = 1.739; *l*_As–O2_ = 1.752
As(III)–C_20_H_36_N_2_O_8_ **(c)**	3	11.88	− 7.99	− 7.71	0.29	*l*_As–N1_ = 1.929; *l*_As–N2_ = 1.976

Chemical hardness of chemical species characterizes their resistance towards electron cloud polarization or deformation. It was introduced by R.G. Pearson^36^ is an important indicator of chemical stability. According to maximum hardness principle, hard molecules are more stable compared to soft ones. Some recent applications of this principle were presented in detailed in the book edited by Islam and Kaya^37^. According to Table 1, the structure presented in Fig. 2b is harder and more stable compared to others structures. There is an inverse relation between chemical hardness and polarizability. According to the minimum polarizability principle, “in a stable state, the polarizability is minimized.” This principle also supports the stability of geometry presented in Fig. 2b. Another indicator of the chemical stability is the electrophilicity index. Minimum electrophilicity principle proposed via Parr’s electrophilicity index states: “in a stable state, electrophilicity is minimized.” Electrophilicity values calculated in the light of Parr’s electrophilicity index are equal to 111, 14.45 and 220 eV, for geometries presented in Fig. 2a–c, respectively. Therefore, the predictions from the minimum electrophilicity, maximum hardness and minimum polarizability principles are in good agreement with each other. All these principles recognized the structure presented in Fig. 2b as the most stable complex among all studied geometries. The adsorption mechanism regarding to this study is as in the Fig. 2b. Calculated values of electro donating and electro accepting powers for this configuration were equal to 0.64 and 4.41 eV, respectively.

In the light of chemical hardness concept introduced by R.G. Pearson^36^, the nature of the chemical interactions, reactivity and stability of chemical species can be illuminated. According to chemical structure of the considered PMaema molecule, it is a hard base. Hard and Soft Acid–Base (HSAB) principle implies classification of Lewis acids and bases into hard and soft. According to this classification, As(III) ion belongs to hard acids (see the Table 1 of Ref.^36^). According to HSAB principle, the electrostatic interaction between hard acids and hard bases should be quite strong. This is why the interaction between As(III) ion and PMaema molecule is powerful.

### Selection of elution solvent type

The function of the eluent is to transfer the analyte adsorbed on the solid phase into the final solution. However, eluent should not deform the solid adsorbent. We investigated nine different eluents to select the best of them. The recoveries of As(III) ions with using of acetone, THF, acetonitrile, sulfuric acid, water, nitric acid, hydrochloric acid, methanol and ethanol as the eluent were equal to 45.7%, 51.8%, 59.5%, 68.9%, 74.1%, 75.9%, 81.0%, 82.5% and 91.7%, respectively. The highest recovery for As(III) ions was achieved in the presence of ethanol. This is why ethanol was chosen as the preferable eluent for further optimization.

### Chemometric approaches

#### Statistical analysis

To achieve an efficient and fast extraction of As(III) ions from the selected samples, the effect of experimental parameters (pH, PMaema amount, adsorption time and ethanol volume) were investigated with the Box-Behnken design. We assumed second-order polynomial dependence of the goal function on experimental parameters and derived corresponding regression coefficients. We also used analysis of variance (ANOVA) for statistical investigation of the significance of each regression coefficient. Based on data presented in Table 2, we obtained quadratic polynomial dependence of recovery on experimental parameters. This dependence includes linear, binary and quadratic terms:

$$Recovery \, \left( \% \right) \, = 90.12 - 4.20 \, A \, + 8.52 \, B \, + 9.86 \, C + 11.05 \, D \, - 3.20 \, AB \, + 1.40 \, AC \, - 6.20 \, AD \, + 9.57 \, BC - 3.60 \, BD \, + 4.95 \, CD \, - 18.35 \, A^{2} - 13.24 \, B^{2} - 14.97 \, C^{2} - 18.38 \, D^{2}$$

**Table 2:** Regression coefficients and ANOVA analysis of the quadratic model calculated with the stepwise method.

Source	Sum of squares	df	Mean square	F-value	p-value	
Model	9652.55	14	689.47	84.65	< 0.0001	Significant
**Linear interaction**						
A	211.68	1	211.68	25.99	0.0001	
B	872.11	1	872.11	107.07	< 0.0001	
C	1166.24	1	1166.24	143.18	< 0.0001	
D	1465.23	1	1465.23	179.88	< 0.0001	
**Binary interaction**						
AB	40.96	1	40.96	5.03	0.0405	
AC	7.84	1	7.84	0.9625	0.3421	
AD	153.76	1	153.76	18.88	0.0006	
BC	366.72	1	366.72	45.02	< 0.0001	
BD	51.84	1	51.84	6.36	0.0234	
CD	98.01	1	98.01	12.03	0.0034	
**Square interaction**						
A^2^	2310.00	1	2310.00	283.60	< 0.0001	
B^2^	1202.34	1	1202.34	147.61	< 0.0001	
C^2^	1536.01	1	1536.01	188.57	< 0.0001	
D^2^	2316.30	1	2316.30	284.37	< 0.0001	
Residual	122.18	15	56.79			
Lack of fit	76.77	10	7.68	0.8454	0.6175	Not significant
Pure error	45.41	5	9.08			
Cor total	9774.73	29				
Std. dev	2.85	R^2^		0.9875		
Mean	64.14	Adjusted R^2^		0.9758		
C.V. %	4.45	Predicted R^2^		0.9481		
		Adeq precision		27.1523		

The values of R^2^ and adjusted-R^2^ (0.9875 and 0.9758, respectively) confirm reliability and predictive power of this regression. AVONA analysis suggests the significance of the established design: *F*-value and *p*-value were equal to 84.65 and < 0.0001, respectively. We also calculated the “lack-of-fit” value, which is used in ANOVA to evaluate the significance and authenticity of the established design. The calculated “lack-of-fit” value was equal to 0.6175, much more than the critical value 0.05. This was the additional justification of the validity of proposed regression.

#### Assessment of significant factors

**Figure 3:**
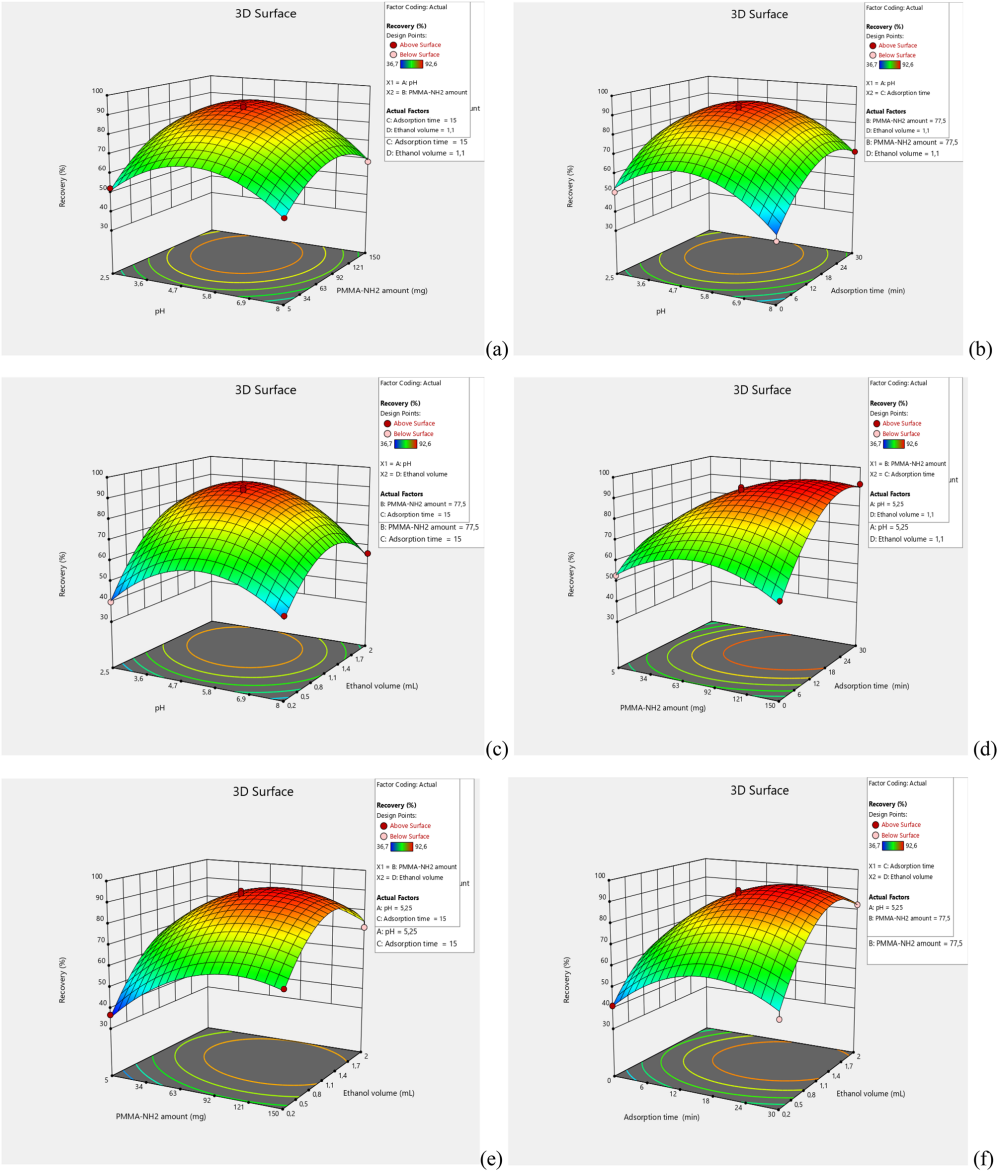
(**a**–**f**) Three-dimensional plot for determination of As(III). (**a**) pH-PMaema amount (mg); (**b**) pH- adsorption time (min); (**c**) ethanol volume (mL)-pH; (**d**) adsorption time (min)- PMaema amount (mg); (**e**) ethanol volume (mL)- PMaema amount (mg); (**f**) adsorption time (min)- ethanol volume (mL).

The 3D response surface plots of As(III) recovery depending on experimental factors are presented in Fig. 3a–f. The adsorption of the analyte to the synthesized PMaema is strongly depends on the pH of the sample solution. The PMaema could hydrolyze in strong acid or alkali. As a result, the recovery of As(III) could be decreased. Therefore, the pH of sample solution was variated in 2.5 to 8.0 range to provide the best preconcentration and determination of the As(III) ions. The pH 4.3 resulted in the highest recovery, see Fig. 3a–c. The amount of PMaema in the sample solution must be sufficient to ensure complete adhesion of As(III) ions on the PMaema. Figure 3a, d and e show the increase of the recovery with the amount of PMaema, if this amount is lower than 110 mg. Further increase of the amount results to decrease of the recovery. Therefore, 110 mg is the optimal amount of PMaema. Adsorption time should be sufficient to complete the adsorption of As(III). We varied the adsorption time in 0 to 30 min range; the highest recovery was achieved at 22 min (see Fig. 3b, d and f). Longer times was not change recovery significantly. The volume of the eluent (ethanol) should be sufficient to provide transforming of the As(III) ions adsorbed on the PMaema into the solution phase. Too low volumes of ethanol does not provide complete desorption, whereas too high volumes result in both low concentration of the As(III) in solution phase and deformation of the solid adsorbent. We varied the ethanol volume in the range 0.2–2.0 mL (see Fig. 3c, e, f). The maximal recovery of the As(III) ions was achieved at 1.5 mL. Therefore, it is the optimal value of ethanol volume.

#### Optimum conditions

Analytical data obtained as a result of the application of the experimental model were evaluated with the help of the statistical program (BBD design). In this evaluation, the statistical program was commanded to achieve the highest recovery for As(III). In the light of this command, the most suitable values for the variables optimized by the statistical program were produced as follows. The pH, PMaema amount, adsorption time and eluent volume were 4.3, 110 mg, 22 min and 1.5 mL, respectively. The estimated recovery value for As(III) at the optimum conditions selected by the statistical program was 96.69% with desirability of 1.0. In three-replicate experimental studies conducted at selected optimum conditions, the resulting recovery for As(III) were 96.2%, 96.7%, and 95.4%, respectively. For these studies, standard deviation values ranged from 0.8 to 1.2%. It is easily understood from the results that there is a high agreement between the experimental data and the estimated values of the statistical model. As a result, the above values for As(III) ions with high recovery and good fit were chosen as optimized values for the relevant variables in our study.

### Reusability of PMaema

Generally, the cost of newly synthesized PMaema directly depends on its reusability. Analysis costs for routine experiments reduces significantly, if the synthesized adsorbent can be used repeatedly. We investigated the reusability of the PMaema copolymer using model solutions contained 40 ng L^−1^ of As(III) under the optimized conditions. As a result of this study, the adsorption feature of the PMaema copolymer was determined to be apparently stable (< 5%) after the sequential application of > 15 cycles of adsorption and desorption of the As(III) ions.

### Effect of sample volume

The effect of sample volume on the recovery of the As(III) ion should be investigated to calculate the preconcentration factor (PF) of the proposed method using the optimum conditions. By definition, the PF is the ratio of sample volume to final volume (1.5 mL). We varied the sample volume in the range 10–350 mL, whereas the concentration of As(III) was kept to be equal to 40 ng L^−1^. It has been observed that the As(III) ions were quantitatively recovered when the sample volume was less than 150 mL. Therefore, 150 mL was adopted as the optimal sample volume. Corresponding value of the PF is equal to 100.

### Sorbent capacity

Sorbent capacity was defined as the maximum amount of the analyte retained by one gram of the solid adsorbent. We used the batch adsorption method to determine the sorbent capacity of the PMaema copolymer. In our experiment, 75 mg of the sorbent was added to 50 mL of the model solution containing As(III) ions under the optimum conditions. The mixture was stirred for 20 min to guarantee the achievement of equilibrium. The aqueous portion was then separated by decantation, and the amount of As was measured by HGAAS. The capacity of the sorbent was calculated according to the following formula;

1 $${\text{Q}}_{{\text{e}}} = \, \left( {{\text{C}}_{{\text{i}}} - {\text{C}}_{{\text{e}}} } \right){\text{ V W}}^{{ - {1}}}$$

where Qe was the sorbent capacity (mg g^−1^), Ci and Ce were the initial and final amounts (mg L^−1^) of As(III) ions, W (g) was the amount of the adsorbent and V (mL) was the volume of the model solution. The maximum sorbent capacity was 75.8 mg g^−1^. This value indicates that the PMaema copolymer possesses a strong adsorption capability toward the As(III) ions.

### Interfering ions

Interfering ions can affect the steps of adsorption of As(III) ions to the PMaema copolymer and cause reducing its sorption efficiency. This was why we tested selectivity of the PMaema using 30 mL of the analyte with model As(III) concentration (40 ng L^−1^) in the presence of some cations and anions commonly present in the real samples. The tolerance limit was calculated from the ratio of the ions amount, which results in ± 5% relative error in the analytical signal, to the amount of analyte. The recoveries values and tolerance limits are presented in Table 3. The result proves that the interfering ions have no significant effect on the recoveries of As(III) ions.

**Table 3:** Tolerance limits of interfering ions for determination of 40 ng L^−1^ As(III) after application method.

Interfering ions	Tolerance limit^a^	Recovery (%)	RSD (%)
K^+^	1500	99.2	2.9
HPO_4_^2−^	1500	98.5	2.6
SCN^−^	1500	98.6	3.1
SO_4_^2−^	1000	97.9	2.8
C_2_O_4_^2−^	1000	98.1	2.9
F^−^	1000	98.4	2.7
I^-^	1000	98.8	3.1
Zn^2+^	750	98.2	2.9
Mo^6+^	750	97.9	2.7
Sb^3+^	750	97.8	2.5
Mn^2+^	500	97.0	2.8
Se^4+^	500	97.4	2.8
Ni^2+^	500	96.8	2.7
Pb^2+^	500	96.6	3.1
Co^2+^	500	97.1	3.4
As^5+^	250	96.2	3.0
Al^3+^	250	96.4	3.3
Fe^3+^	100	95.7	3.5
Mn^3+^	100	95.1	3.6

^a^[Interfacing ions]/[As(III)].

### Analytical performance

Analytical performance characteristics such as linearity, limit of detection (LOD), limit of quantification (LOQ), enrichment factor (EF), relative standard deviation (RSD%) and recovery were investigated under the optimum conditions defined above. These characteristics were determined according to the IUPAC recommendation. The linearity of calibration curve was confirmed from 10 to 500 ng L^-1^ with coefficients correlation R^2^ = 0.9956. The LOD and LOQ were calculated as 3s_blank_/m and 10s_blank_/m, respectively (s_blank_ denotes the standard deviation of the blank solutions, and m stands for the slope of the linear section of the calibration curve). LOD and LOQ were equal to 3.3 ng L^−1^ and 10 ng L^−1^, respectively. The EF, calculated as the ratio of the slopes of calibration curve before and after microextraction, was equal to 85. The recoveries (spiked at 50 ng L^−1^) remained in the range of 91.5–98.6% with acceptable RSD% less than 4.3%. Comprehensive results were summarized in Table 4.

**Table 4:** Analytical performance characteristics.

Characteristics	After microextractionn	Before microextraction
Regression equation	A = 0.0513[As(III). ng L^−1^] + 0.0255	A = 0.0036[As(III). ng L^−1^] + 0.0016
Correlation coefficient (R^2^)	0.9956	0.9914
Linear range (ng L^−1^)	10–500	750–2500
LOD (ng L^−1^)	3.3	227
LOQ (ng L^−1^)	10	750
Recovery (%)	91.5–98.6	85.9–94.3
RSD (%)	4.3	5.6
PF	100	–
EF	85	–
Sorbent capacity (mg g^−1^)	75.8	–

### Accuracy and precision

The precision was investigated by determining the As(III) in the added rice sample at low (25 ng L^−1^), middle (150 ng L^−1^), and high (300 ng L^−1^) amounts with four repetitions of the sample solution each day. The repeatability was obtained by analyzing the selected rice sample four times during one day, while the reproducibility was obtained by analyzing the selected rice sample four times a day over three consecutive days. The RSDs% for repeatability and reproducibility analysis were lower than 3.6% and 4.1%, respectively. The obtained results were given in Table 5.

**Table 5:** Determination of the As in two standard reference materials after application method (N:5).

SRMs	Certified value (µg kg^−1^)	Determined (µg kg^−1^)	Recovery (%)	t-test^a^	F-test^a^
1573a (tomato leaves)	112.6 ± 2.4	110.4 ± 3.5	98.0	1.44	2.13
1568a (rice flour)	290 ± 30	281 ± 12	96.9	1.67	4.67

^a^Theoretical value for t- and F-values for 5 degrees of freedom and 95% confidence limits are 2.57 and 6.39 respectively.

In order to assess the accuracy of the optimized method, two standard reference materials (SRMs) such as SRM 1573a (Tomato leaves) and SRM 1568a (Rice flour) were analyzed. The obtained results (see Table 6) revealed no significant difference at 95% confidence confirming the accuracy of the method. On the other hand, the precision of the method was validated by applying *F*-test and *t*-test at a 95% confidence level. In all cases, the *F*_exp_ and *t*_exp_ were found lower than the *F*_theoretical_ and t _theoretical_.

**Table 6:** Repeatability and reproducibility studies for the precision of the application method.

	Repeatability (N:4)			Reproducibility (N:4 × 3)		
	Low	Middle	High	Low	Middle	High
RSD (%)	3.5	3.3	3.9	3.7	4.1	4.0
Recovery (%)	94.1	96.9	98.3	92.8	95.6	97.1

### Analytical applications

In the present study, the total amount of inorganic arsenic was determined as the equivalent of As(III) in the selected samples. In this context, the reduction study of As(V) to As(III) was conducted according to the reported in the literature^38^. A 1.0 mL of solution containing 0.75 g potassium iodide and 1.25 g ascorbic acid was added to 30 mL of sample solutions. To complete reduction, the resulting mixture was left at room conditions for 30 min. After the reduction of As(V), the proposed method was applied for the preconcentration and determination of total inorganic arsenic in foods (rice, brown rice, carrot, pepper, flour, milk powder, tomato, cabbage, garlic, chicken liver and fish) and water samples (tap water, well water, river water, waste water, and bottled water) with a standard addition approach. Analytical results obtained from the analysis of food samples were presented in Table 7. Arsenic could not be detected in two selected food samples including milk powder and garlic. The highest arsenic content was detected in brown rice (114.8 µg kg^−1^). Recovery values were ranged from 94.2 to 103.2%. The RSDs% were less than 3.7%. These values demonstrate the applicability of the method and independence of the results on matrix effect of the foods.

**Table 7:** Results of HGAAS analysis of food samples spiked with known amounts of As(III).

Sample	Spiked	Determined (µg kg^−1^)	Recovery (%)	RSD (%)
Rice	–	96.7	–	2.4
	100	192.1	95.4	2.5
Brown rice	–	114.8	–	3.1
	100	216.9	102.1	3.4
Carrot	–	2.7	–	3.2
	100	99.5	96.8	2.8
Pepper	–	54.8	–	2.6
	100	152.5	97.7	2.7
Flour	–	33.6	–	2.1
	100	136.8	103.2	3.3
Milk powder	–	ND^a^	–	3.5
	100	97.5	97.5	3.7
Tomato	–	65.9	–	2.9
	100	160.9	95.0	2.6
Cabbage	–	4.2	–	2.5
	100	102.5	98.3	3.1
Garlic	–	ND	–	3.0
	100	103.7	103.7	2.8
Chicken liver	–	85.9	–	2.6
	100	180.1	94.2	3.1
Fish	–	74.2	–	2.6
	100	169.5	95.3	2.9

^a^Not determined.

All water samples were through 0.22 μm cellulose membrane filters (Millipore) prior to their microextraction procedure. analytical results including the recovery and RSDs% in different waters were given in Table 8. While no arsenic was detected in bottled water, tap water and river water, 45.1 ng L^−1^ and 98.5 ng L^−1^ arsenic were determined in well water and wastewater, respectively. Recovery for all water samples was in the range 95.6–103.7% with RSD lower than 3.4%.

**Table 8:** Results of HGAAS analysis of water samples spiked with known amounts of As(III).

Sample	Spiked (ng L^−1^)	Determined (ng L^−1^)	Recovery (%)	RSD (%)
Tap water	–	ND^a^	–	3.2
	50	47.9	95.8	3.3
	100	97.6	97.6	3.0
Well water	–	45.1	–	2.4
	50	93.7	97.2	2.7
	100	143.1	98.0	2.9
River water	–	ND		2.6
	50	48.1	96.2	2.8
	100	98.5	98.5	3.1
Waste water	–	125.6	–	3.0
	50	173.4	95.6	3.2
	100	222.5	96.9	3.4
Bottled water	–	ND	–	2.5
	50	51.2	102.4	2.7
	100	103.7	103.7	3.0

^a^not determined.

### Method performances comparison

Performance of the proposed method was compared with some of the previously reported analytical techniques. As can be seen from Table 9, compared to the same HGAAS determination with different microextraction procedure like CPE, DLLME, DES-VAME, our method provides higher PF, lower LOD and wider linearity. This may be due to the high selectivity of the synthesized PMaema amphiphilic copolymer toward As(III) ions and performed BBD optimization. Note that we took into account binary and square interactions of variables, which are commonly ignored in univariate optimization. Our method possessed satisfactory sorbent capacity and lower RSD% in comparison with the most others methods. Other significant advantages of the proposed method were its low cost as well as rapid and simple separation.

**Table 9:** Comparison of the proposed method with other methods applied for preconcentration and determination of inorganic arsenic.

Sample	Microextraction procedure	Detection method	Linearity (ng L^−1^)	LOD (ng L^−1^)	RSD (%)	Sorbent capacity (mg g^−1^)	EF or PF	References
Food	CPE	HG-AFS	550–20,000	170	9.3		10.9	^2^
Rice	SPME	FI-HG AAS	1200–10,000	40	5.5	–	17	^50^
Water	SPME	GFAAS	1–20,000	87	4.5	15	13	^51^
Water	SPME	ICP-OES		150	4.3	55	–	^52^
Rice	CPE	ETAAS	50–10,000	10	2.5	–	73.8	^53^
Rice	MAS-LIS-DLLME	GFAAS	40–5000	5	3.7	–	10	^54^
Food and water	DES-VAME	HG AAS	15–150	7.5	2.7	–	85	^55^
Food and water	DES-UA-LPME	ETAAS	–	10	4.3	–	25	^38^
Food and water	SPME	HG-AAS	10–500	3.3	4.3	75.8	85/100	This study

*SPME* solid phase microextraction, *FI-HG AAS* Flow injection-hydride generation atomic absorption spectrometry, *GFAAS* graphite furnace atomic absorption spectrometry, *ICP-OES* inductively coupled plasma-optical emission spectrometry, *CPE* cloud point extraction, *ETAAS* Electrothermal atomic absorption spectrometry, *MAS-LIS-DLLME* fully-automated magnetic stirring-assisted lab-in-syringe dispersive liquid–liquid microextraction, *DES-VAME* deep eutectic solvent based vortex assisted microextraction, *DES-UA-LPME* deep eutectic solvent ultrasound-assisted liquid phase microextraction, *HG-AFS* hydride generation atomic fluorescence spectrometry.

## Discussion

In summary, a new synthesized amphiphilic copolymer with pendant primary amine groups (PMaema) was prepared as solid-phase adsorbent. We used PMaema for the preconcentration of As(IIII) ions from the food samples and aqueous solution. Box–Behnken design was performed to optimize the experimental conditions. After microextraction, the amount of arsenic in the samples was measured through HGAAS technique. The adsorption of As(III) ions to the synthesized PMaema was also supported using computational chemistry studies. According to the solid phase adsorbent principle, the application of the amphiphilic copolymer during the adsorption and desorption stages can obviously improve the recovery of As(III) ions. The optimized procedure was successfully applied to detect inorganic arsenic in the real samples, and displayed some advantages such as high sorbent capacity, cheapness, acceptable sensitivity, high precision, simplicity and environmental friendliness. Therefore, we consider that the optimized procedure is a competitive alternative for determining ultra-trace amounts of arsenic in real samples.

## Materials and methods

### Apparatus and software

The ultra-level of arsenic was determined by Hydride Generation Atomic Absorption Spectrometry (HG-AAS, Shimadzu AAS-6300 model, Kyoto, Japan). Measurement parameters of the HGAAS were operated lamp current at 10 mA, wavelength set at 197.2 nm, slit width set at 0.2 nm and air/acetylene flame at 4.66 L min^−1^. The more detailed conditions were shown in Table 10. In the phase separation process, a centrifuge (320-Model Hettich Universal, Darmstadt, Germany) was applied. The vortex VG3 model (IKA GmbH, Germany) and microwave digestion (Milestone Ethos Easy Advanced, Italy) were utilized for desorption step and preparation of food samples, respectively. The pH of the working solutions was adjusted using a pH-meter (JP Selecta, Barcelona, Spain). Ultra-pure water with a resistivity of 18.2 MΩ was used in all experiments and was obtained from an Milli-Direct Q3 purification system (Millipore, Bedford, MA, USA). All optimization studies were conducted in triplicate and all results were averaged. Design-Expert trial version 12.0.1. (Stat-Ease Inc., Minneapolis) was used to generate the Box–Behnken design^39^. ^1^H NMR spectrum of the copolymer (PMaema) was taken at 25 °C with an Agilent 600 MHz Nuclear Magnetic Resonance (NMR) (Agilent, Santa Clara, CA, USA) spectrometer equipped with a 3 mm broadband probe. FTIR spectrum of the copolymer was recorded using Perkin-Elmer Spectrum 100 Fourier Transform Infrared (FTIR) spectroscopy (Perkin-Elmer Inc., Norwalk, CT, USA).

**Table 10:** Optimal parameters of HG-AAS for the measurement of arsenic.

Flame conditions	Arsenic	
Wavelength (nm)	197.2	
Lamp current (mA)	10	
Spectral bandwidth (nm)	0.2	
Temperature of quartz tube (°C)	900	
Air–acetylene flame (L min^−1^)	7.0	
Gas flow rates (L min^−1^)	1.5	

Hydride generation conditions	Operating range	Optimized value
1.0% (w/v) of NaBH_4_ volume. mL	0.1–3	1.5
5.0 mol L^−1^ of HCl volume. mL	0.1–3	1.0
Argon flow rate. mL min^−1^	50–200	120

### Reagents

Stock solution (1000 mg L^−1^) of As(III) was prepared by dissolving the appropriate amount of Na_3_AsO_3_ (Sigma, St Louis, MO, USA). The calibration and working solutions were prepared by appropriate stepwise dilution of the stock solutions in 1.0% (w/v) of HCl solution. Ethanol, methanol, acetone, nitric acid, acetonitrile, hydrochloric acid, sulfuric acid, and tetrahydrofuran (THF) were purchased from Merck (Darmstadt, Germany). Methyl methacrylate (MMA), 2-aminoethyl methacrylate (aeMA) and 2,2′-azo bis isobutyronitrile (AIBN) used for synthesis of copolymers were purchased from Sigma-Aldrich (St. Louis, MO, USA). Acetate buffer solution (pH 4.3) was prepared with the appropriate mixture of sodium acetate and acetic acid in the water**.** To validate the data obtained from experimental measurements, two standard reference materials (SRMs) such as SRM 1573a (Tomato leaves) and SRM 1568a (Rice flour) were employed^3^.

### Sample collection

The applicability of the study was tested on food and water samples^3^. Food samples including rice, brown rice, carrot, pepper, flour, milk powder, tomato, cabbage, garlic, chicken liver and fish were collected randomly from local markets in Sivas, Turkey. Tap water was supplied from our laboratory. Bottled water was purchased from the market in Sivas, Turkey. Well water was collected from agricultural land in Sivas. The river water was collected from the surface of the Kızılırmak river passing through Sivas. The waste water was collected from the industrial area in Sivas.

### Microwave digestion

The selected food samples and SRMs were prepared by closed-vessel microwave digestion^40^. At first, concentrated HNO_3_ (6 mL) and concentrated H_2_O_2_ (2 mL) were added to 0.3 g of the samples in Teflon vessel and immediately placed in the microwave digestion system. The mixture solution was applied to a 2-step power (W) controlled program with a final step of 550 (W) (200 °C) for 10 min. After microwave digestion, the resulting solution were cooled and then transferred to polyethylene bottles. Finally, the obtained solution was completed to 100 mL with ultrapure water.

### Synthesis of poly(methyl methacrylate-co-2-aminoethyl methacrylate copolymer (PMaema)

A mixture of methyl methacrylate (MMA, 2.12 g), 2-aminoethyl methacrylate (aema, 4.03 g) and 2,2′-azo bis isobutyronitrile (AIBN, 0.016 g) was dissolved in 5 mL of DMF under argon^34^. The solution was kept at 80 °C for 60 min in an oil bath. Then, it was precipitated from distilled water (200 mL). Polymer recovered was washed with distilled water several times and dried under vacuum at 40 °C for 2 days. Yield was 4.06 g. PMMA homopolymer was synthesized using the same procedure with MMA monomer and AIBN only. Poly2aema homopolymer was synthesized using the same procedure with aema monomer and AIBN only.

### Atomic model of PMaema

**Figure 4:**
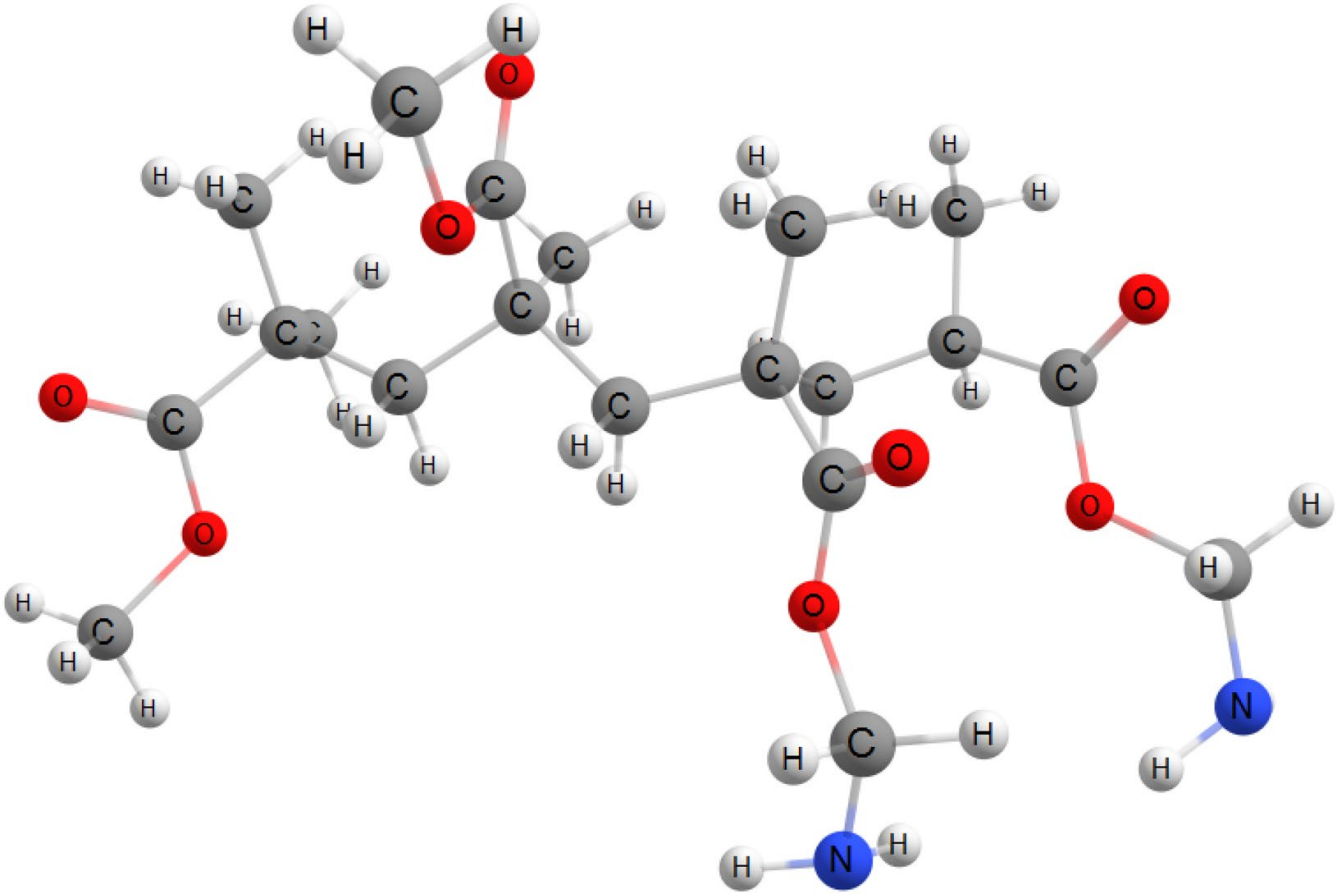
Optimized geometry of the C_20_H_36_N_2_O_8_ molecule used as a model of PMmae polymer chain.

We considered molecule C_20_H_36_N_2_O_8_ as a model of PMaema polymer chain. The molecule includes four fragments; two of them are functionalized by NH_2_ moieties^41^. Ends of the molecule are terminated by hydrogens to avoid dangling bonds. Optimized structure of the molecule was presented in Fig. 4. Note that the fragments with NH_2_ moieties are almost parallel to each other, whereas two fragments without NH_2_ form an obtuse angle.

### Box–Behnken design

Experimental designs are widely used to reduce the number of optimization studies. Here, Box–Behnken design (BBD) based on response surface methodology (RSM) was continued with 30 combination runs of four experimental variables and 6 randomly distributed center points. The experimental variables included in the BBD were pH (A), PMaema amount (B), adsorption time (C) and ethanol volume (D), with each variable investigated at 3 levels comprising of low (− 1), mid. (0) and high (+ 1). The obtained model and results were given in Table 11. The mathematical relationship of the response (as absorbance) on the experimental variables can be approximated by the second order equation^42^:

2 $${\text{y = b}}_{{0}} { + }\mathop \sum \limits_{{\text{i = 1}}}^{{\text{k}}} {\text{b}}_{{\text{i}}} {\text{x}}_{{\text{i}}} { + }\mathop \sum \limits_{{\text{i = 1}}}^{{\text{k}}} {\text{b}}_{{{\text{ii}}}} {\text{x}}_{{1}}^{{2}} { + }\mathop \sum \limits_{{{1} \le {\text{i}} \le {\text{j}}}}^{{\text{k}}} {\text{b}}_{{\text{ij }}} {\text{x}}_{{\text{i}}} {\text{ x}}_{{\text{j}}} { }$$

**Table 11:** Factors their symbols levels design matrix and results in the BBD to evaluate the microextraction method.

Factors		Symbol	Levels of factors		
			Low	Middle	High
pH		A	2.5	5.25	8
PMaema amount (mg)		B	5	77.5	150
Adsorption time (min)		C	0	15	30
Ethanol volume (mL)		D	0.2	1.1	2

Run	*A*	*B*	*C*	*D*	Recovery (%)
1	5.25	150	0	1.1	52.6
2	8	77.5	30	1.1	64.4
3	5.25	77.5	30	0.2	47.1
4	5.25	150	15	2	71.5
5	8	77.5	15	2	55.9
6	5.25	77.5	15	1.1	90.8
7	5.25	77.5	30	2	82.6
8	8	77.5	0	1.1	38.1
9	2.5	5	15	1.1	52.3
10	5.25	77.5	15	1.1	92.1
11	8	5	15	1.1	49.3
12	5.25	150	30	1.1	91.5
13	2.5	150	15	1.1	74.4
14	5.25	5	15	2	62.2
15	2.5	77.5	30	1.1	71.1
16	2.5	77.5	15	2	74.6
17	5.25	77.5	15	1.1	89.4
18	5.25	77.5	0	2	56.8
19	8	150	15	1.1	58.6
20	5.25	5	30	1.1	53.4
21	2.5	77.5	0	1.1	50.4
22	5.25	77.5	15	1.1	91.4
23	5.25	77.5	0	0.2	41.1
24	2.5	77.5	15	0.2	39.8
25	5.25	77.5	15	1.1	84.4
26	8	77.5	15	0.2	45.9
27	5.25	150	15	0.2	60.4
28	5.25	5	15	0.2	36.7
29	5.25	77.5	15	1.1	92.6
30	5.25	5	0	1.1	52.8

where y was the absorbance; *Xi and Xj* were variables (i and j ranged from 1 to k); *b*_*0*_ was constant term; *b*_*i*_ was linear coefficient, *b*_*ii*_ were interaction coefficient, and _bjj_ was quadratic coefficient; k was number of independent variables (k = 4 in this study).

### Details of density functional calculations

All calculations were performed with B3LYP exchange-corrected functional and 6–311 ++ G[2d,2p] electronic basic set^36^. We used the GPU-based TeraChem software^43^. Geometry optimizing was carried out with the efficient geomeTRIC energy minimizer^44^. To take into account non-covalent interactions, the dispersion corrections D3 proposed by Grimme^45^ were included. Solvent effects were introduced in the frame of the COSMO solvent model^46^ implemented in TeraChem. The dielectric constant of the solvent (acidic ethanol) was chosen to be equal to 25.3. Chemical reactivity descriptors such as chemical potential (µ), electronegativity (χ), hardness (η) and softness (σ) were calculated using ground state ionization energy (I) and electron affinity (A). The relations between total electronic energy (E), number of electrons (N), ionization energy and electron affinity and quantum descriptors mentioned above were given via the following equations;

3 $$\mu = - \chi = \left[ {\frac{\partial E}{{\partial N}}} \right]_{\nu (r)} = - \left( {\frac{I + A}{2}} \right)$$

4 $$\eta = \left[ {\frac{{\partial^{2} E}}{{\partial N^{2} }}} \right]_{\nu (r)} = \frac{I - A}{2}$$

5 $$\sigma = 1/\eta$$

The *I* and *A* values can be evaluated from frontier orbitals energies in accordance with the Koopmans theorem. Parr et al.^47^ proposed the electrophilicity index *ω* based on electronegativity and absolute hardness values of chemical species. Chattaraj defined the nucleophilicity index *ε*. These indexes were calculated as

6 $$\omega = \chi^{2} /2\eta$$

7 $$\varepsilon = 1/\omega$$

In recent years, Gazquez et al.^48^ introduced electrodonating power (ω^−^) and electroaccepting powers (ω^+^). These parameters predict electron donating and electron accepting capabilities of molecule, respectively. They were calculated from *I* and *A* with the following equations:

8 $$\omega^{ + } = \left( {I + 3A} \right)^{2} /\left( {16\left( {I - A} \right)} \right)$$

9 $$\omega^{ - } = \left( {3I + A} \right)^{2} /\left( {16\left( {I - A} \right)} \right)$$

### Optimized solid phase microextraction

The experimental steps of the optimized SPME method were carried out as follows. Initially, 110 mg of the PMaema was exactly weighed, and added to 30 mL of sample solution containing 40 ng L^−1^ of As(III) into a 50 mL-centrifuge tube. Then, acetate buffer solution was added to the solution to obtain pH of 4.3. The resulting mixture was kept on an orbital shaker for 22 min to accelerate the mass transfer of the As(III) from the sample solution to the solid adsorbent. The extracted As(III) on the PMaema was separated from the sample solution by centrifugation (4000 rpm, 5 min), and subsequently aqueous phase was emptied by Pasteur pipette. Next, in order to elute the adsorbed As(III) on the PMaema, 1.5 mL of ethanol (as an eluent solvent) was added to the remaining solid phase. Finally, the amount of arsenic in eluted solution was determined by HG-AAS^49^. The sample blanks were prepared in the same manner. All experiments were repeated three times.
